# Functional Habitude

**Published:** 1882-07

**Authors:** L. C. Ingersoll

**Affiliations:** Keokuk


### THE DENTAL REGISTER.

Vol. XXXVI.] JULY, 1882. [No. 7.

### FUNCTIONAL HABITUDE.

BY L. C. INGERSOLL, KEOKUK.

(Read before the Iowa State Dental Society, May 3, 1882.)

There is no remark more trite than that "we are creatures of habit;" and no fact more generally recognized. Taken in connection with another fact, *that we are susceptible of change*, we have two facts of very common occurrence, for explanation of which we must carefully study our physical natures.

An individual man is an aggregation of organic functions. When we use the personal pronoun *I* or *he* it is the most concise possible mention of this aggregation of human functions. Yet a single letter *I* has in it this enlarged meaning. It is doubtful that we carry this enlarged meaning into the proverb above mentioned, though we express it in five words. We ordinarily speak of the individual man in his unity—not in his complexity. The common observer studies man synthetically—seldom analytically. Yet this analysis of human functions is the only true method of study.

When we say of a man that he is a creature of habit, do we mean man in his unity, or in his complexity? Do we mean man considered synthetically, or analytically?

It will be my purpose in this paper to illustrate the fact that habit pertains to every organ and function of the human body; that bodily organs and functions are subject to training into new habits and modes of action. Then to draw some plain and practical conclusions:

Walking is, perhaps, the most familiar illustration of a muscular habit. Through much perseverance the infant acquires it, and thus enters the second period of life—childhood. He *walks* into it. In infancy the attempt to walk engaged all the powers of the expanding mind, and held in abeyance every voluntary function and muscle of the body. When the little creature makes its first attempts to walk, it is vain for any one to try and catch its eye, or provoke a smile. It can do nothing else but walk. But when the habit of walking is gained, the child can walk and talk, can laugh, look, and handle, as though not walking at all. This is the result of the muscles being trained into a habit of action without the mind's attention and direction.

The same is true of the muscular action of the fingers in playing the piano. Before the muscular habit is formed the movement of each individual finger must receive the direction and training of the mind, and the attempt to do anything else, or think of anything else, is distracting. But when the habit is gained, the fingers move as unconsciously as the feet in walking.

We are familiar also with the facts concerning the sense of smell, and the sense of taste also, that they change, and that the stomach is susceptible to training.

Food that at first is so nauseating as to be expelled from the stomach, may, by continuance of its use, not only be tolerated in the stomach, but become nutritious. Tobacco, which at first acts as the most nauseating poison, reversing all the pleasurable functions of the body, by continuance of its use, trains the functions into being pleasantly excited by it. So with alcohol, opium, arsenic, and other poisons, however disgusting or destructive in their first effects on normal functions, may, by continued use, become almost harmless. This change of function may affect even the structure of the tissue itself.

A wart is a familiar example of this change of tissue. Some cause operating at a single point on the skin, at length, changes the functional habit of the skin, so that instead of producing normal tissue, it produces an excrescence.

The slight irritation of a heavy tobacco pipe resting on the lip, together with the irritating effect of the nicotine of tobacco, will, in some cases, so modify and change the tissue of the lip that a cancerous tumor will grow.

Acclimation, on removing to a distant country, is another familiar example of change in the functional habitude. At first the functions may be impaired by change of location, but afterward they become trained into harmony with the new environment. This explains why so many persons finding health in a new climate return to their former homes only to return to their former condition. The mistake is in not remaining long enough for the functions to take on the new habit, and there is a ready return of the impaired functional performance.

There is a typical form of every organ and tissue of the body to which the forces of nature, acting under the laws of organic life, are continually tending. So, also, there is a complete and perfect functional performance toward which nature tends. This is the *natural* habit of the body, and any deviation from this, whether for a brief time, or habitually, is disease. Disease may be structural, functional, or both. There cannot be structural lesion and perfect performance of function. On the other hand, there may be imperfect performance of function without structural lesion. A change of function may be for a longer or shorter time, and the organs form no new habit of action, but readily return by a natural tendency to a healthy condition. This deviation from nature we term primary or acute disease. Chronic disease exists when this changed condition is continuous, and the organs form new and fixed habits. A cure involves a recognition of the susceptibility of the functions to change.

Bodily functions and organs are not self-acting, with few exceptions. They are but organized powers subject to influences from without, which act upon them as excitants. Hence the air, the sunlight, the sounds that come to our ears, the objects that greet the eye, the odors that give to us the sense of smell, the food that enters the stomach, all combine to excite and keep in motion our bodily functions. If there is perfect adaptation and harmony of all these influences with our bodily functions, we have perfect health, provided, of course, that the organization be complete.

In the discipline and training of functions regard must be had for the proper relation of natural excitants to the powers of the function. Natural excitants may by increase or decrease of quantity be changed to irritants and endanger the organism which they are adapted to nourish. Too much food exhausts the powers of digestion. Too much light irritates the eye instead of giving it healthful exercise of vision. An odor, however delightful, may, in excess, become digusting to the sense of smell.

Were the changes thus induced, inevitable, were there no possible relief by a compensating change of bodily function, we can conceive how a slight cause might eventuate in death. If the natural warmth of the body were increased to fever heat without the possibility of adjustment by a change of these functions the body would be consumed by the heat. A beam of light thrown into the eye with such intensity as to cause pain, if continued without change and adjustment of the eye to the excess of light, would undoubtedly cause death. This susceptibility of the organism to change and the power of adjustment to uncongenial surroundings is the law of life which protects the organism from destruction from slight causes. A force at once overpowering destroys life; but a force not overpowering at first, may be warded off, and its immediate tendency and effect thwarted by adaptation to the new processes going on.

I think I have now sufficiently illustrated the principle to make it plain. Yet I wish to guard against a mistake in its application. A notion has prevailed that things which are hurtful to the body in its normal condition cease to be so by the continued use of the poison. Or, as we say, we become used to it so that we no longer notice its effects. That change which is effected by the use of tobacco is a change from a normal or healthy condition to one that is abnormal. It can in no sense be said that nature accepts the new condition induced by the hurtful practice of drinking intoxicating beverages because the dilirium which they tend to produce is to some extent overcome by the organs and functions becoming adjusted to them. The delirium is simply changed to some other effect less dangerous. If the delirium continued, nature must succumb. But by a modification of the effects, nature *tolerates* the drug.

The body politic never accepted slavery as the normal condition of man in such a sense as that by so doing, it ceased to be an evil. But government sought to so far ward off and modify the monster evil that it could be *tolerated.*

Society does not accept drunkenness as a good thing because men are used to it. But society does seek to protect itself against the *enormity* of the evil to such an extent as to *tolerate* the evil while it allows it to exist. If it continues unchecked and with no compensating adjustments, the evil would become wholly intolerable.

So our animal organism *tolerates* the presence of disease without accepting the abnormal condition as the healthful condition.

I will now proceed to some practical conclusions:

The first is, the folly of trusting to any one medicine for a great length of time to produce a desired effect. Sometimes when medicine is prescribed the patient remarks, "I have worn out that medicine."

In malarial districts there are persons who have used quinine till it no longer has its usual effect. A man who has used opium for its sedative and soporific effects finds at length that these effects are not produced, but by a change of functional action, exhilaration is produced by the same drug which at first induced sleep. Hobbyists who find a "sure cure" in some compound of their own making, for a specific disease, are doomed to a failure at the point of nature's *toleration* of the drug. Thus a remedy which we have trusted for years at length surprises and disappoints us in its results.

Take as an example the use of arsenic for devitalizing the pulp of a tooth. In the normal condition in which a pulp is found, on immediate or recent exposure, arsenious acid applied will produce devitalization in a very few hours without noticable pain. But when by long exposure the functions of the pulp have been modified and adjusted to new conditions, we find cases that will resist the action of arsenic for several days even though often applied. Here, not the arsenic, but other causes have induced the functional change. In such cases the pulp usually dies with a struggle.

This leads me to notice the effect of exposure in changing the functions and in some cases the structure of the pulp. When, by exposure, the air comes in contact with the pulp, it, at first, induces severe pain. But by continued exposure it adjusts itself to the new condition and will tolerate, without painful disturbance, the presence of air and even cold and warm drinks, which at first could not be indulged in without causing pain. This explains the reason why patients so frequently visit a dental office for the purpose of having a tooth filled, saying, "The tooth used to ache, but the nerve is now dead and it does not pain me any more."

The point to be specially noticed is that under certain untoward influences the pulp changes its character, its functional habitude, so as to bear without pain or sensible impression what in its normal condition it would not tolerate—and that this toleration sometimes extends even to the presence of arsenic. Could such a pulp be restored to its normal condition, outward influences would again effect it as before.

This principle explains to us the nature of chronic disease. I would define a chronic disease as an abnormal performance of function which has become so fixed that it has no longer any natural tendency to return to the proper condition. Take the example of pulp disease to which I have already referred. When the pulp of a tooth is first exposed to the atmosphere and fluids of the mouth, severe pain is induced. This we term acute disease; and it has this peculiarity, that when the air and fluids are excluded from the cavity of decay by any simple means, as by a pellet of cotton or wax, the pain ceases and the pulp returns to health. But when by continued exposure the abnormal condition has become fixed in a new and unwonted functional habit, the pulp exhibits no tendency, by exclusion of the air, to return to the normal state. In the acute form of the disease a simple removal of the cause is usually sufficient treatment. In the chronic form of the disease cause, the functional habit, into which it has fallen by long time exposure, must be changed before normal functional performance will be induced. We may have both chronic and acute disease in the same organ at the same time. I will again illustrate by pulp disease. In the chronic form, to which I above alluded, the pulp manifests no diseased condition from mere exposure to the atmosphere and in the ordinary conditions of the mouth. But when food is forced down into the cavity, pain is excited immediately. This is a new influence which the pulp will not tolerate. It now exhibits the character of acute disease. When the pressure is removed, by cleaning the cavity of the foreign substance, the pain ceases and the pulp returns to its chronic and painless condition of abnormity.

It is quite possible that the principle illustrated in this paper, will explain the fact that some diseases seldom, if ever, afflict the body but once. All the functions of the body susceptible to influence by sporadic germs of disease may become so modified and changed in their habitude as not to again return to their natural habit. We may thus, perhaps, answer the question why all persons equally exposed to small pox or measles do not take the disease. Few, if any, persons are found who are in a normal condition of bodily functions. If we could know the physical system perfectly we should find it in every conceivable abnormal condition; some with a functional habitude just fitted to repel the attack of a prevailing disease, and others with a natural or acquired functional habitude just suited to invite the disease. The same explanation may be given for the effects of vaccination—driving the system into such a form of changed functions as to overcome its susceptibility to contagious influences. We are accustomed to esteem vaccination a very wonderful and inexplicable operation. But if we can refer it to this general law of functions it becomes no more wonderful than the changed habit of function, induced by the use of tobacco, or the change induced by the use of opium—such a change that the organs and functions of the body are no longer affected by it as at first.

The susceptibility of the functions of the body to permanent change, explains the fact why the victim of intoxication seldom regains his normal condition. The voluntary functions of the body which normally were under the control of the will-power, and are no longer susceptible to the influence of the will. They have fallen into a new habit. This new functional habitude is a disease in a chronic form, and partakes of the nature of other abnormal actions that have gained a fixedness and permanence. Intoxication should, therefore, be treated medically as well as morally. The disease is one of both body and mind, and while it requires the persuasive power of principle to regain the functions of the will, it requires the persuasive power of medicine to regain the functions of the body.

When the cause of an acute disease is removed we are in the habit of saying that it cures itself, or that nature performs the cure; that is, the cure arises from sources within the body, and is found in this natural tendency before alluded to, *i.e.*, of functions to return to their normal habit. But we find that chronic diseases do not thus cure themselves. The curative power is not from within the body, because this natural tendency to return to normal performance of function has been overcome by a new functional habitude tending perpetually to an abnormal state. The case requires, therefore, some force from without the body to cause the functions to return to their normal condition. This is the theory of medication: in acute disease, to remove the cause; in chronic disease, to change the habit of bodily functions. When the wearing of a plate of artificial teeth has irritated and inflamed the mucous membrane of the mouth to a painful condition, it may be cured by a simple removal of the plate. But when, by continuance of the irritation, a painless tumefaction has been produced, it may be well, for the time being, to remove the cause, but this alone does not effect a cure. The knife and caustics must take a part.

I have attempted to illustrate a principle of our physical natures of very wide application in explaining phenomena which have been esteemed mysterious. What we call freaks of nature, or anomalies, become fewer in proportion as we understand natural laws. All things in nature are wonderful until we understand them. Like the tricks of a juggler, all are inexplicably wonderful until we understand them; then they are only wonderful in their simplicity.

